# Supplementary material for: Topological layer Hall effect in two-dimensional type-I multiferroic heterostructure
Source: Nat Commun. 2025 Jul 3;16:6141. doi: 10.1038/s41467-025-61514-6 (PMC12229577; doi:10.1038/s41467-025-61514-6)
Supplement: Supplementary file 1 — Supplementary Information [file 41467_2025_61514_MOESM1_ESM.pdf]

Supplementary Information

Topological Layer Hall Effect in Two-Dimensional Type-I Multiferroic Heterostructure

Wenhui Du [1], Kaiying Dou [1], Xinru, Li [1], Ying Dai [1,*], Zeyan Wang [1,*], Baibiao Huang [1], Yandong Ma [1,*]

[1] School of Physics, State Key Laboratory of Crystal Materials, Shandong University, Shandanan Street 27, Jinan 250100, China

*Corresponding author: daiy60@sina.com (Y.D.); wangzeyan@sdu.edu.cn (Z.W.); yandong.ma@sdu.edu.cn (Y.M.)

Supplementary Note 1: Calculation details of magnetic parameters in spin Hamiltonian.

The magnetic parameters are obtained from DFT calculations.

DMI parameters: The DMI vector $\mathbf{D}_{ij}$ for the nearest-neighboring Cr atoms can be expressed as $\mathbf{D}_{ij} = d_{\parallel}(\mathbf{z} \times \mathbf{u}_{ij}) + d_{\perp}\mathbf{z}$ with $\mathbf{z}$ being the out-of-plane unit vector and $\mathbf{u}_{ij}$ being the unit vector pointing from site *i* to *j*.

To get the in-plane component $d_{\parallel}$, four different spin-spiral configurations are considered, as shown in Supplementary Fig. 4. The energy of for spin configurations represented by E_1, E_2, E_3 and E_4 can be written as:

$$E_1 = E_0 + \frac{3}{2} d_{\parallel}^{\mathrm{top}} |\mathbf{S}|^2 \times 4$$

$$E_2 = E_0 - \frac{3}{2} d_{\parallel}^{\mathrm{top}} |\mathbf{S}|^2 \times 4$$

$$E_3 = E_0 + \frac{3}{2} d_{\parallel}^{\mathrm{bot}} |\mathbf{S}|^2 \times 4$$

$$E_4 = E_0 - \frac{3}{2} d_{\parallel}^{\mathrm{bot}} |\mathbf{S}|^2 \times 4$$

Therefore, the in-plane component $d_{\parallel}$ of top and bottom layer can be obtained by $d_{\parallel}^{\mathrm{top}} = \frac{E_1 - E_2}{12}$ and $d_{\parallel}^{\mathrm{bot}} = \frac{E_3 - E_4}{12}$.

Heisenberg exchange interaction parameters: To calculate exchange interaction parameters, we consider eight different spin configurations as show in Supplementary Fig. 10. The energy of different spin configurations can be written as:

$$E_1 = E_0 - 12J_1^{\mathrm{top}} - 12J_2^{\mathrm{top}} - 12J_1^{\mathrm{bot}} - 12J_2^{\mathrm{bot}} - 4J_1^{\mathrm{inter}} - 24J_2^{\mathrm{inter}}$$

$$E_2 = E_0 - 12J_1^{\mathrm{top}} - 12J_2^{\mathrm{top}} - 12J_1^{\mathrm{bot}} - 12J_2^{\mathrm{bot}} + 4J_1^{\mathrm{inter}} + 24J_2^{\mathrm{inter}}$$

$$E_3 = E_0 + 4J_1^{\mathrm{top}} + 4J_2^{\mathrm{top}} + 4J_1^{\mathrm{bot}} + 4J_2^{\mathrm{bot}} - 4J_1^{\mathrm{inter}} + 8J_2^{\mathrm{inter}}$$

$$E_4 = E_0 + 4J_1^{\mathrm{top}} + 4J_2^{\mathrm{top}} + 4J_1^{\mathrm{bot}} + 4J_2^{\mathrm{bot}} + 4J_1^{\mathrm{inter}} - 8J_2^{\mathrm{inter}}$$

$$E_5 = E_0 - 12J_1^{\mathrm{top}} - 12J_2^{\mathrm{top}} + 4J_1^{\mathrm{bot}} + 4J_2^{\mathrm{bot}}$$

$$E_6 = E_0 - 4J_1^{\mathrm{top}} + 4J_2^{\mathrm{top}} - 12J_1^{\mathrm{bot}} - 12J_2^{\mathrm{bot}}$$

$$E_7 = E_0 + 4J_1^{\mathrm{top}} + 4J_2^{\mathrm{top}} - 12J_1^{\mathrm{bot}} - 12J_2^{\mathrm{bot}}$$

$$E_8 = E_0 - 4J_1^{\mathrm{top}} + 4J_2^{\mathrm{top}} - 4J_1^{\mathrm{bot}} + 4J_2^{\mathrm{bot}} + 4J_1^{\mathrm{inter}} + 8J_2^{\mathrm{inter}}$$

Based on the above formula, intralayer and interlayer exchange interaction parameters can be obtained.

Test calculations confirm that both the anisotropic symmetric exchange (−0.006 meV) and second-nearest-neighbor DMI (0.002 meV) in $CrInSe_3$ are exceptionally weak, validating their exclusion from our spin Hamiltonian.

Single-ion anisotropy interaction: The single-ion anisotropy K is calculated as: $K = E_{100} - E_{001}$, where E_{100} and E_{001} represent the energies when the magnetization axis of a given layer is aligned along the in-plane and out-of-plane directions, respectively.

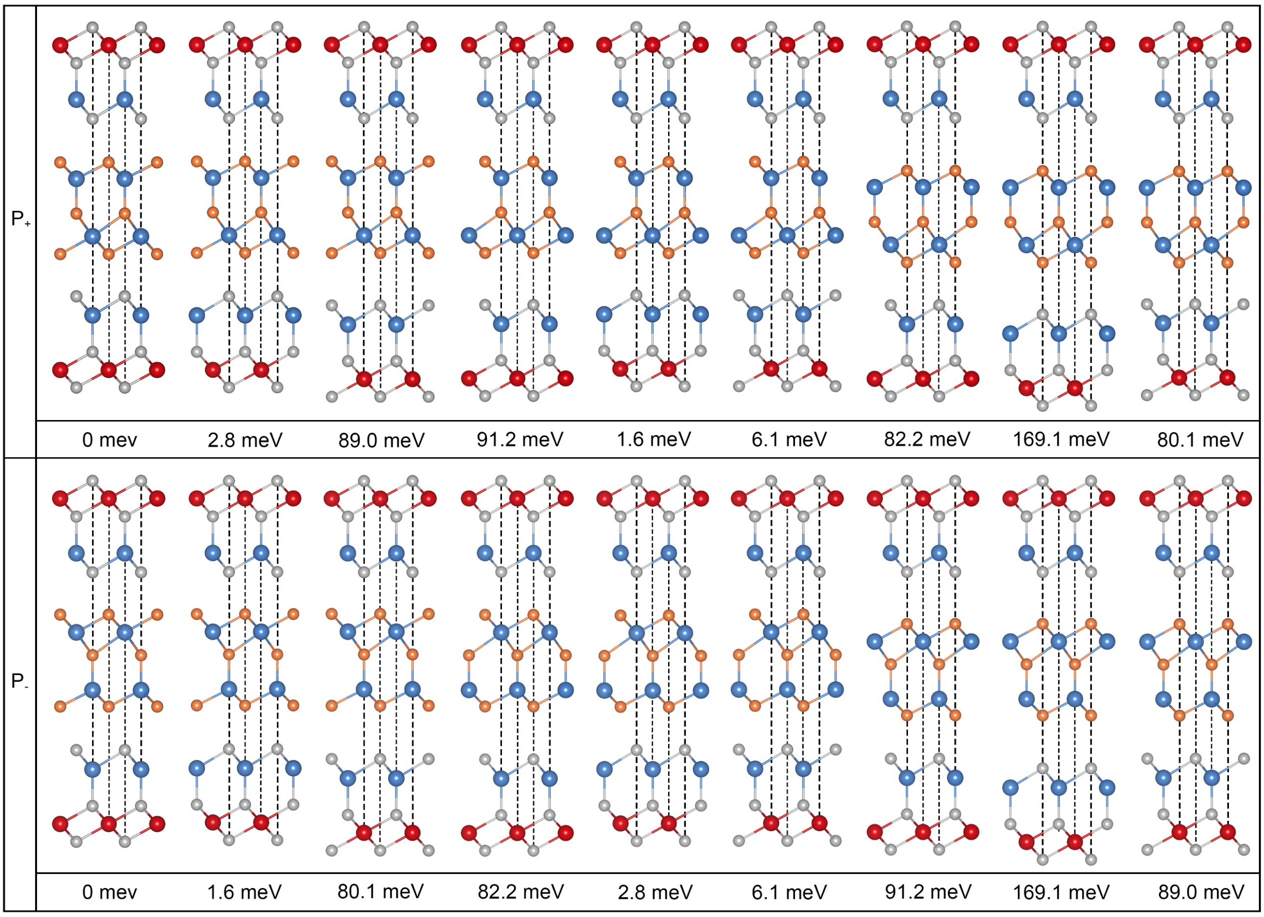

Supplementary Fig. 1. Crystal structures of $CrInSe_3/In_2S_3/CrInSe_3$ heterostructure under different stacking configurations and total energy differences per unit cell between the energetically most stable structure and other stacked configurations for $CrInSe_3/In_2S_3/CrInSe_3$ heterostructure.

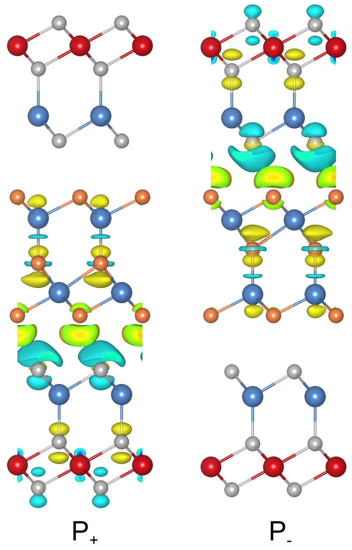

Supplementary Fig. 2. Differential charge densities of $CrInSe_3/In_2S_3/CrInSe_3$ heterostructure under P_+ and P_- phases.

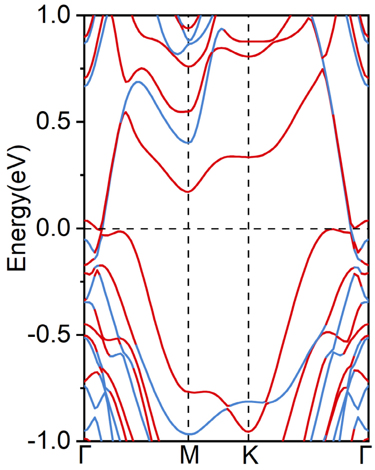

Supplementary Fig. 3. Band structure of $CrInSe_3/In_2S_3/CrInSe_3$ heterostructure with considering SOC. The red and blue lines correspond to spin-up and spin-down states, respectively.

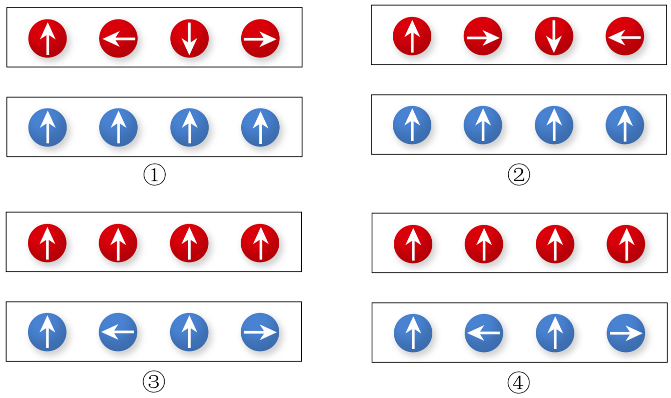

Supplementary Fig. 4. Different spin-spiral configurations used to obtain in-plane component $d_{\parallel}$. Red/blue balls represent the magnetic atoms of top/bottom layer. White vectors indicate the directions of magnetic moments.

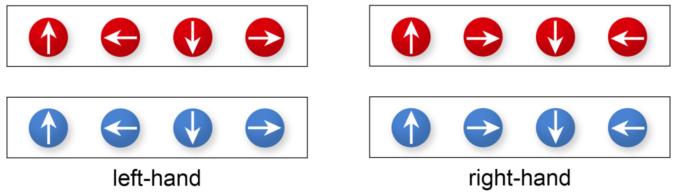

Supplementary Fig. 5. Left and right-hand spin-spiral configuration used to obtain atomic-layer-resolved SOC energy difference ΔE_{soc}. Red/blue balls represent the magnetic atoms of top/bottom layer. White vectors indicate the directions of magnetic moments.

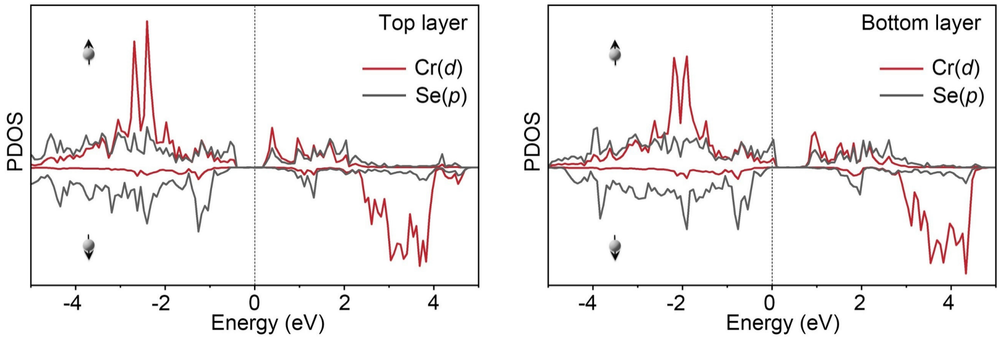

Supplementary Fig. 6. Projected density of states of Cr and Se atoms in top and bottom $CrInSe_3$ layers for P_+ phase.

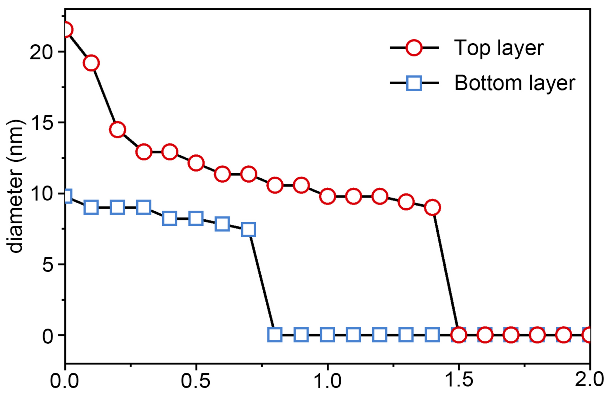

Supplementary Fig. 7. Skyrmion diameters for P_+ phase of $CrInSe_3/In_2S_3/CrInSe_3$ heterostructure as a function of magnetic field.

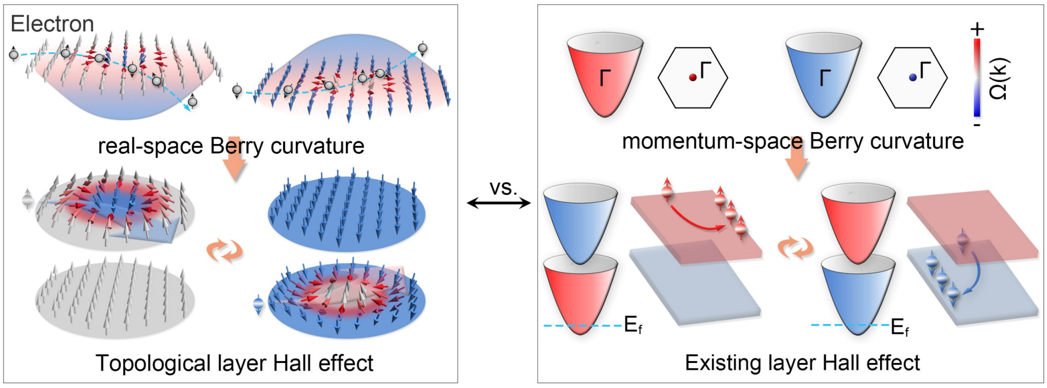

Supplementary Fig. 8. Conceptual diagram comparing the underlying mechanisms of the topological layer Hall effect and existing layer Hall effect.

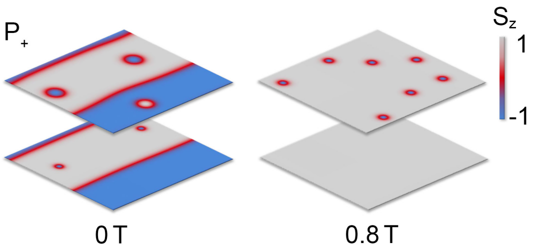

Supplementary Fig. 9. Spin textures in (P_+, M_+) phase of $CrInSe_3/In_2S_3/CrInSe_3$ heterostructure with considering the third nearest-neighbouring exchange interaction.

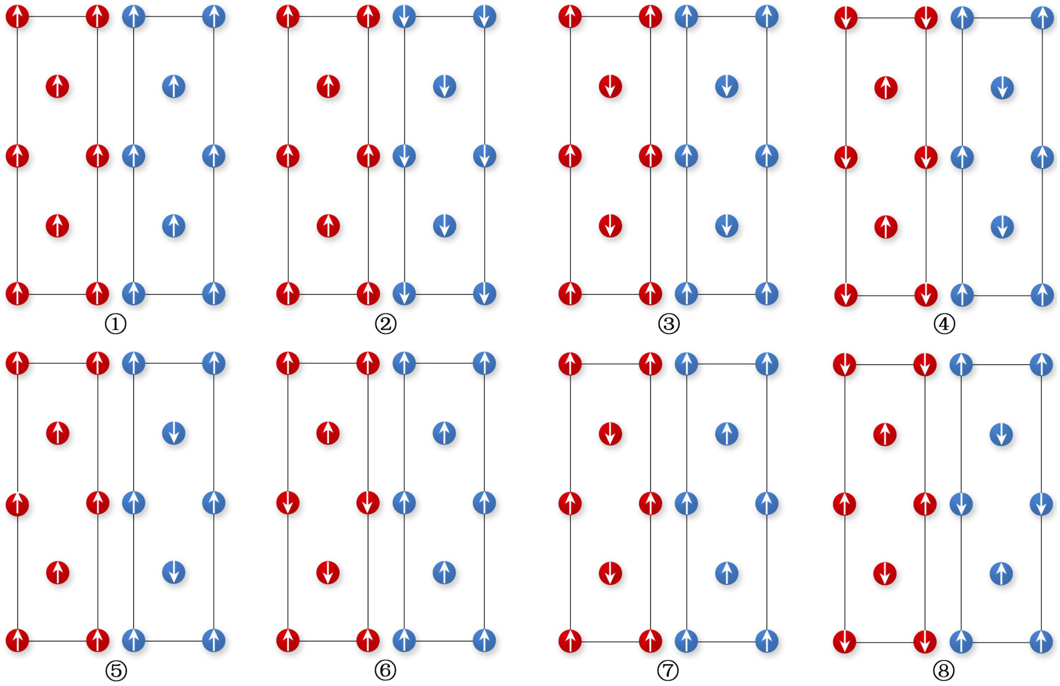

Supplementary Fig. 10. Different spin configurations used to obtain Heisenberg exchange interaction parameters. Red/blue balls represent the magnetic atoms from top/bottom layer. White vectors indicate the directions of magnetic moments.

Supplementary Table 1. Exchange interaction parameters (in meV) for P_+ phase with third-nearest-neighbor terms considered.

J_1^{top}	J_2^{top}	J_3^{bot}	J_1^{bot}	J_2^{bot}	J_3^{bot}	J_1^{inter}	J_2^{inter}	J_3^{inter}
25.606	0.973	-1.533	26.790	1.074	-1.468	0.003	0.002	0.002
